# New Trends in Episodic Memory Assessment: Immersive 360° Ecological Videos

**DOI:** 10.3389/fpsyg.2018.01878

**Published:** 2018-10-02

**Authors:** Silvia Serino, Claudia Repetto

**Affiliations:** ^1^Department of Psychology, Catholic University of the Sacred Heart, Milan, Italy; ^2^Applied Technology for Neuro-Psychology Laboratory, Istituto Auxologico Italiano, Milan, Italy

**Keywords:** ecological validity, virtual reality, memory, 360° videos, assessment

## Abstract

How best to measure memory in a reliable and valid way has been intensely debated in neuropsychological literature. Specifically, classical neuropsychological tests often fail to predict real-life performance or capture the multifaceted nature of memory function. To solve these issues, there has been a growing emphasis on the use of more ecological memory assessment. In this scenario, several virtual reality based tools have been developed to evaluate memory function. The aim of the current perspective is to discuss critically the possibilities offered for episodic memory assessment by one of the most innovative trends in the technology field, i.e., 360° videos. Immersivity, egocentric view and realism appear to be crucial features of 360° videos enabling them to enhance the ecological validity of classical assessment tools of memory abilities.

## The State-Of-The Art in Memory Assessment

How best to measure memory in a reliable and valid way has been intensely debated in neuropsychological literature. Classical neuropsychological tests have proved to have an adequate clinical predictive value. However, the correlation between these tests and subjective memory complaints and/or everyday memory functioning appears to be quite inadequate (see for example, Chaytor and Schmitter-Edgecombe, 2003; Reid and Maclullich, 2006). The majority of neuropsychological tests (including memory assessment) have been developed following a “construct-driven approach”; starting from a solid theoretical paradigm, they evaluate abstract constructs without any reference to the real-life performance or behavior (Parsons, 2015; Parsons et al., 2017). In the last 20 years, however, there has been a growing emphasis on the use of a more ecological neuropsychological assessment. This is the so-called “function-led approach,” that implies the direct observation of behavior for the definition of a more ecologically valid neuropsychological testing (Parsons, 2015; Parsons et al., 2017). A representative example of this approach in the memory domain is the Rivermead Behavioural Memory Test (RBMT; (Wilson et al., 1986, 1989), which includes a series of daily-life tasks (i.e., recognize a picture, remember an appointment, encode, and store a route, etc.) to evaluate memory abilities in people following brain injury. Beyond the issue of ecological validity, neuropsychological tests appear unreliable in capturing the “complexity” of memory functioning (and its components). For instance, episodic memory has been traditionally defined as the memory of personally experienced events in their spatiotemporal context along with their perceptual details (Tulving and Murray, 1985; Tulving, 2002). The multiple components of episodic traces (what, when, where, spatial details) are merged through a process known as binding (Kessels et al., 2007), dependent upon the medial temporal lobes (Tulving and Markowitsch, 1998; Eichenbaum, 2000). So far, episodic memory assessment is usually conducted in clinical settings by asking older adults to remember a verbally presented story (Spinnler and Tognoni, 1987) or a list of words (Delis et al., 1987). Consequently, classical memory tests usually evaluate the memory components in isolation, thus failing to offer a global picture of the true essence of episodic retrieval (Tulving, 2002).

In this context, virtual reality (VR) appears to be a suitable technology for a function-led assessment of cognitive functioning (Rizzo et al., 2004; Bohil et al., 2011; Parsons, 2015; Parsons et al., 2017). Indeed, VR allows the development of a new class of ecologically valid neuropsychological tools able to simulate real-life situations. Moreover, this approach permits advantages in terms of time consumption, economic cost, precise control over the delivery of stimuli, and safety (Bohil et al., 2011). A growing body of VR-based tools has been developed to specifically evaluate episodic memory functions (Burgess et al., 2001; Spiers et al., 2001; Parsons and Rizzo, 2008; Plancher et al., 2008, 2012; Repetto et al., 2016; Parsons and Barnett, 2017; Picard et al., 2017). Within virtual environments, participants can be immersed in digital scenarios that represent everyday situations (i.e., a kitchen, a park, a museum); in these highly ecological situations, simple tasks can easily be implemented to evaluate the multifaceted nature of episodic memory.

For example, Burgess et al. (2001) exploited the potentiality of VR to investigate the neural basis of episodic recollection. In this study, participants were immersed in a highly ecological virtual town to follow a route where they received 16 objects from two people in two places. During a functional neuroimaging resonance, the memory of the objects, places and people was tested. A further example was offered by Piolino’s team, who developed a VR-based test for assessing the different components of episodic memory (Plancher et al., 2008, 2010). Participants were immersed in a virtual town (with either passive or active navigation), where they encountered various episodes (i.e., a man playing a guitar, a barking dog, etc.) to be remembered in their specific spatiotemporal context.

Furthermore, within virtual environments, it is possible to make the episodic memory task more complex and closer to the real life situation by adding intervening executive tasks (Parsons and Barnett, 2017); for example, Parsons and Barnett (2017) asked participants to shop for a list of items in a virtual grocery, ignoring items that were not present on this list as well as other irrelevant announcements.

However, the assessment of episodic memory in virtual environments has already been tested in clinical settings. Plancher et al. (2012) carried out a study involving healthy participants, amnesic mild cognitively impaired patients, and Alzheimer’s patients. One of the goals was to benchmark the virtual-reality episodic memory task against traditional neuropsychological tools for the assessment of episodic memory and subjective memory complaints. The virtual test correlated better with subjective memory complaints than classic memory tests did; importantly, the virtual task was also able to characterize the different cognitive profiles of the three populations, thanks to its suitability in capturing the multi-componential nature of the episodic memory.

To further enhance the ecological validity of neuropsychological testing and to deeply evaluate the multiple aspects of episodic memory, one possibility is offered by one of the most innovative trends in the technology field, i.e., 360° videos.

## The Potential Use of 360° Videos for Memory Investigation

Videos with 360 degrees are spherical videos recorded by special cameras with omnidirectional lenses, able to collect images from all the way around the space. Usually 360° videos are recorded in natural life settings and displayed through a head-tracked Head Mounted Display (or on a low-cost cardboard viewer connected to a smartphone provided with a gyroscope). During the playback, a user can control the viewing direction by means of moving the head in a very realistic way (looking up one can see the sky/roof; looking down, one can see the floor/ground; and if one wants to see what happens on the left/right, it is sufficient to turn the head accordingly, as in real life situations). Therefore, these videos offer the viewers the ability to feel inside the represented environment and be the protagonist of action that unfolds before their eyes (Broeck et al., 2017). Lastly, it should be noted that no advanced programming skills are required to customize 360° videos; thus they can easily and economically be adopted in clinical settings without specific technical effort.

For these capabilities, 360° videos are often considered a special kind of VR, even though several features of 360° videos differ from graphic-based VR (Slater and Sanchez-Vives, 2016), as will be discussed later in this section.

Because of better availability and technological advancement of the necessary equipment (i.e., omnidirectional camera and HMD/cardboard viewers), the use of 360° videos is rapidly growing in several fields, such as immersive journalism, brand advertisement, and live sports. Many online platforms and social networks, such as YouTube, Facebook, and Vimeo, are now ready to support these videos, thus contributing to their sharing among the general public. Conversely, little effort has been put forward at a scientific level to understand the cognitive processes underlying this special 360° visual experience, or its capabilities for the investigation or assessment of cognitive abilities such as memory.

A recent study in this direction has been presented by Robertson et al. (2016) who used 360° videos to investigate how visual memory for panoramas are formed in the brain. In two behavioral experiments, authors asked participants to study novel 360° panoramic environments representing real urban scenes of a city, actively explored by means of a VR headset. Crucially, the study conditions varied in relation to the type of visual stimulus presented: in the naturalistic condition, the scenes were seamless and displayed continuously, exactly as they had been recorded from the real environment; in the morph condition, the scenes included pictures from different environments, gradually morphed within the main panorama. Results of the first study (study 2 in the research report) indicated that associative memory was higher for scenes studied in the naturalistic condition than in the morph condition. The second experiment (study 4 in the research report) specifically assessed the impact of perceptual priming (a screenshot of the studied panorama vs. a black screen) on memory for the correct position of a scene image. Again, the perceptual priming effect was superior in the naturalistic condition. All in all, these findings demonstrated that, even at the peripheral stage of visual processing (i.e., within the retina), individuals experience only discrete fleeting views of the environment; in the brain these images are merged together to create the perceptual experience of a coherent continuous panorama. Interestingly, the recognition of this seamless view has a positive impact on visual memory. These first results on the relationship between the naturalistic view of a panoramic scene and visual memory suggest that 360° videos may be the ideal tool for future investigations of memory processes, as they provide a visual experience very close to the natural visual exploration of the environment. More generally, we posit that 360° videos could effectively address the issue of ecological validity in memory assessment, thanks to several of its features.

The first one is the possibility to experience the environment from an egocentric perspective. According to Rubin and Umanath (2015) a visual perspective is crucial in distinguishing between the process of “remembering” and that of “knowing”: the egocentric view allows one to recollect memories of events and relive them during recall [in fact, this process is often referred to as mental time travel (Tulving, 1983; Suddendorf et al., 2009)]. Importantly, in this view, first-person (e.g., experiencing the scene from one’s own eyes) and third-person perspectives (e.g., experiencing the scene as an observer) are both egocentric in that they place the person remembering the scene relative to the spatial context in which the event occurs. Memory research has underlined that recent autobiographical memories are more likely retrieved from a first-person perspective, whereas older memories are more often associated with an observer perspective (Nigro and Neisser, 1983; Rice and Rubin, 2009). While recording the 360° videos, the experimenter could choose the egocentric view he/she is interested in: placing the camera over one’s head creates the illusion of being the protagonist of the scene (first-person perspective); conversely, placing the camera in a given position within the environment allows the scene to be experienced in the role of external observer (third-person perspective). This flexibility gives the chance to adapt the panoramic video to the actual needs of the researcher, targeting different memory processes.

The second element related to the ecologic validity is the realism. Realism is at least twofold: first, it can be intended as a perceptual similarity to the real world (Lovett et al., 2015; Parsons, 2015); second, it could be referred to as behavioral realism, that is the extent to which a user responds to the represented environment in the same way he/she would respond to the real one (Freeman et al., 2000). As far as perceptual realism is concerned, 360° videos, being recorded in real-life situations, are exact representations of the real world; therefore, they would not suffer from the pitfalls encountered when creating computer-generated virtual environments. With respect to behavioral realism, ergonomic research has demonstrated that a 360° panorama can outperform VR. In particular, Higuera-Trujillo et al. (2017) directly compared the psychological responses evoked by three simulated environmental set-ups (photograph, 360° panorama, and VR) with responses elicited in a real environment. Specifically, authors measured the affective and emotional states triggered by the different environmental set-ups and discovered that 360° panoramas produced results closest to reality. Psychological studies confirmed that 360° videos are able to induce emotions, assessed by both self-reports and psychophysiological parameters. Chirico et al. (2017) administered 360° videos and traditional 2D videos displaying awe-inspiring and neutral content. During the video exposure, they recorded physiological parameters and after the experience, they asked participants to rate the level of awe. Results indicated that panoramic videos enhanced the intensity of awe experienced, compared to 2D videos, and also were able to activate more the parasympathetic system. Similarly, panoramic videos are reported to stimulate the psychophysiological correlates of anger arousal (Macedonio et al., 2007).

The third feature to consider is the immersivity, defined as the possibility to experience the environment from an immersive perspective, while isolated from the real world (Coelho et al., 2006; Diemer et al., 2015). Immersivity is supported by the use of a head-mounted display to view the videos. Immersivity and realism are two of the sub-components of presence. Although there are several slightly different conceptualizations of this construct (Lombard and Ditton, 1997; Riva et al., 2004, 2011; Coelho et al., 2006), presence can be defined as “the sense of being there” in either a virtual or physical environment (Riva and Mantovani, 2012). The capability to induce the sense of presence is highly relevant in the assessment of memory, as it has been demonstrated that the former directly affects the latter (Sutcliffe et al., 2005; Slobounov et al., 2015). Makowski et al. (2017) asked people who had just watched a movie in a cinema to rate the sense of presence and emotional experience during the projection. Crucially, they correlated these self-reports with people’s ability to recall specific factual memories (details of the movie) and temporal memories (order of the scenes) and found that higher levels of presence were associated with better factual memory (but not temporal memory). More importantly, the impact of emotion on memory recall was mediated by presence, confirming its crucial role in memory encoding.

Although 360° videos are as powerful as computer-generated VR in relation to their egocentric view and immersivity, and panoramic videos could even outperform VR in relation to realism, one feature recognized as a strong point in VR is missing in 360° videos: active navigation (Repetto et al., 2016). Indeed, videos do not allow one to choose the direction of navigation, which is defined during recording and cannot be actively changed during the playback. In a computer-generated virtual environment, the user can move in any direction, approach objects and people, and change the path of navigation; however, when watching a 360° video, the user is automatically conducted through the environment and forced to follow the direction of navigation selected during the video recording. Nonetheless, the user can actively explore the environment by moving his/her head and directing attention toward the portion of the 360° visual field he/she is interested in. The active exploration is enhanced if the video is recorded with moving viewports (i.e., the camera moves within the environment while recording) (Broeck et al., 2017). Lastly, a further disadvantage of the 360° video compared to computer-generated VR is the lack of interaction with the elements of the environment.

## Future Directions

**FIGURE 1:**
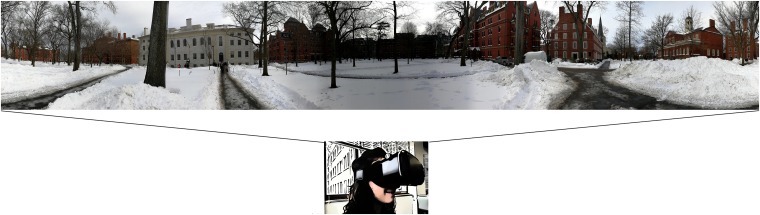
An example of 360° video for episodic memory assessment. The participant, wearing a head-mounted display, is immersed within the 360° scenario and (s)he is free to move his/her head to explore the environment. The participant is prompted to watch the video and remember as much details as possible (about WHAT happens, WHEN it happens, and WHERE it happens). Written informed consent was obtained from the depicted individual for the publication of this image.

As discussed above, immersivity, egocentric view and realism all concur to enhance the ecological validity of a video, and all are involved in episodic memory. As such, we propose that panoramic videos could be used as an assessment tool for episodic memory abilities (see **Figure 1**). In this approach, 360° videos would be the means to present the stimulus to be remembered; similar to more classical assessment methodologies, the outcome measures would be recall of details of the video (the what-when-where components of the episodic recall, as suggested by Tulving, 2002) and recognition of the encoded information. Additionally, the 360° videos also could offer the possibility to collect eye-tracker measures, potentially useful to identify the pattern of visual exploration typical in good and bad memory performers.

These special videos have already been used to evaluate cognitive abilities in a clinical setting, in particular for the assessment of executive function in Parkinson’s disease (Serino et al., 2017). In this study authors administered the 360° version of the Picture Interpretation Test (Rosci et al., 2005) to Parkinson’s disease patients and healthy controls, discovering that this tool correlated with neuropsychological measures of executive functions and was able to reliably discriminate between the two groups (patients vs. healthy controls).

In the same way, panoramic videos could be employed for the assessment of episodic memory in other populations, such as elderly and psychiatric patients. The memory assessment of the elderly is particularly important for detection of early memory decline and the identification of degenerative disorders: indeed, an ecological memory evaluation (by administering videos representing real-life scenarios) could help identify those subtle amnestic deficits that usually escape formal memory assessment, yet account for the subjective memory complaints (Balash et al., 2013) sometimes reported by the elderly. Furthermore, it has been proposed that episodic and autobiographical memory impairments documented in some psychiatric diseases could be due to a specific deficit in scene construction, that is, “*the process of mentally generating and maintaining a complex and coherent scene or event. This is achieved by the retrieval and integration of relevant informational components, stored in their modality-specific cortical areas, the product of which has a coherent spatial context, and can then later be manipulated and visualized*” (Hassabis and Maguire, 2007, p. 299). Some authors have investigated this construct by asking patients to imagine personal life situations (Raffard et al., 2010), but the possibility to immerse patients in real-life scenes, targeting specific emotions, could be much more helpful in providing objective and controllable clues to memory performance.

## Author Contributions

SS and CR conceived and wrote the paper.

## Conflict of Interest Statement

The authors declare that the research was conducted in the absence of any commercial or financial relationships that could be construed as a potential conflict of interest.

## References

[B1] Balash Y. Mordechovich M. Shabtai H. Giladi N. Gurevich T. Korczyn A. D. (2013). Subjective memory complaints in elders: depression, anxiety, or cognitive decline? *Acta Neurol. Scand.* 127 344–350. 10.1111/ane.12038 23215819

[B2] Bohil C. J. Alicea B. Biocca F. A. (2011). Virtual reality in neuroscience research and therapy. *Nat. Rev. Neurosci.* 12 752–762. 10.1038/nrn3122 22048061

[B3] Broeck M. Van den Kawsar F. Schöning J. (2017). “It’s all around you: exploring 360° Video Viewing Experiences on Mobile Devices,” in *Proceedings of the 2017 ACM on Multimedia Conference - MM ’17* (New York, NY: ACM Press), 762–768. 10.1145/3123266.3123347

[B4] Burgess N. Maguire E. A. Spiers H. J. O’Keefe J. (2001). A temporoparietal and prefrontal network for retrieving the spatial context of lifelike events. *Neuroimage* 14 439–453. 10.1006/nimg.2001.0806 11467917

[B5] Chaytor N. Schmitter-Edgecombe M. (2003). The ecological validity of neuropsychological tests: a review of the literature on everyday cognitive skills. *Neuropsychol. Rev.* 13 181–197. 10.1023/B:NERV.0000009483.91468.fb 15000225

[B6] Chirico A. Cipresso P. Yaden D. B. Biassoni F. Riva G. Gaggioli A. (2017). Effectiveness of immersive videos in inducing awe: an experimental study. *Sci. Rep.* 7:1218. 10.1038/s41598-017-01242-0 28450730 PMC5430774

[B7] Coelho C. Tichon J. G. Hine T. J. Wallis G. M. Riva G. (2006). “Media presence and inner presence: the sense of presence in virtual reality technologies,” in *From Communication to Presence: Cognition, Emotions and Culture Towards the Ultimate Communicative Experience*, eds Riva G. Anguera M. T. Wiederhold B. K. (Amsterdam: IOS Press), 25–45.

[B8] Delis D. Kramer J. Kaplan E. Ober B. (1987). *California Verbal Learning Test (CVLT) Manual.* San Antonio, TX: Psychological Corporation.

[B9] Diemer J. Alpers G. W. Peperkorn H. M. Shiban Y. Mühlberger A. (2015). The impact of perception and presence on emotional reactions: a review of research in virtual reality. *Front. Psychol.* 6:26. 10.3389/fpsyg.2015.00026 25688218 PMC4311610

[B10] Eichenbaum H. (2000). A cortical–hippocampal system for declarative memory. *Nat. Rev. Neurosci.* 1 41–50. 10.1038/35036213 11252767

[B11] Freeman J. Avons S. E. Meddis R. Pearson D. E. IJsselsteijn W. (2000). Using behavioral realism to estimate presence: a study of the utility of postural responses to motion stimuli. *Presence Teleoper. Virtual Environ.* 9 149–164. 10.1162/105474600566691

[B12] Hassabis D. Maguire E. A. (2007). Deconstructing episodic memory with construction. *Trends Cogn. Sci.* 11 299–306. 10.1016/j.tics.2007.05.001 17548229

[B13] Higuera-Trujillo L. J. Lopez-Tarruella Maldonado J. Llinares M. C. (2017). Psychological and physiological human responses to simulated and real environments: a comparison between photographs, 360° panoramas, and virtual reality. *Appl. Ergon.* 65 398–409. 10.1016/j.apergo.2017.05.006 28601190

[B14] Kessels R. P. C. Hobbel D. Postma A. (2007). Aging, context memory and binding: a comparison of “what, where and when” in young and older adults. *Int. J. Neurosci.* 117 795–810. 10.1080/00207450600910218 17454244

[B15] Lombard M. Ditton T. (1997). At the heart of it all: the concept of presence. *J. Comput. Mediat. Commun.* 3 10.1111/j.1083-6101.1997.tb00072.x

[B16] Lovett A. Appleton K. Warren-Kretzschmar B. Von Haaren C. (2015). Using 3D visualization methods in landscape planning: an evaluation of options and practical issues. *Landsc. Urban Plan.* 142 85–94. 10.1016/j.landurbplan.2015.02.021

[B17] Macedonio M. F. Parsons T. D. Digiuseppe R. A. Weiderhold B. A. Rizzo A. A. (2007). Immersiveness and physiological arousal within panoramic video-based virtual reality. *Cyberpsychol. Behav. Soc. Netw.* 10 508–515. 10.1089/cpb.2007.9997 17711358

[B18] Makowski D. Sperduti M. Nicolas S. Piolino P. (2017). “Being there” and remembering it: presence improves memory encoding. *Conscious. Cogn.* 53 194–202. 10.1016/j.concog.2017.06.015 28676191

[B19] Nigro G. Neisser U. (1983). Point of view in personal memories. *Cogn. Psychol.* 15 467–482. 10.1016/0010-0285(83)90016-6

[B20] Parsons T. D. (2015). Virtual reality for enhanced ecological validity and experimental control in the clinical, affective and social neurosciences. *Front. Hum. Neurosci.* 9:660. 10.3389/fnhum.2015.00660 26696869 PMC4675850

[B21] Parsons T. D. Barnett M. (2017). Validity of a newly developed measure of memory: feasibility study of the virtual environment grocery store. *J. Alzheimer’s Dis.* 59 1227–1235. 10.3233/JAD-170295 28759971

[B22] Parsons T. D. Carlew A. R. Magtoto J. Stonecipher K. (2017). The potential of function-led virtual environments for ecologically valid measures of executive function in experimental and clinical neuropsychology. *Neuropsychol. Rehabil.* 27 777–807. 10.1080/09602011.2015.1109524 26558491

[B23] Parsons T. D. Rizzo A. A. (2008). Initial validation of a virtual environment for assessment of memory functioning: virtual reality cognitive performance assessment test. *Cyberpsychol. Behav.* 11 17–25. 10.1089/cpb.2007.9934 18275308

[B24] Picard L. Abram M. Orriols E. Piolino P. (2017). Virtual reality as an ecologically valid tool for assessing multifaceted episodic memory in children and adolescents. *Int. J. Behav. Dev.* 41 211–219. 10.1177/0165025415616198

[B25] Plancher G. Gyselinck V. Nicolas S. Piolino P. (2010). Age effect on components of episodic memory and feature binding: a virtual reality study. *Neuropsychology* 24 379–390. 10.1037/a0018680 20438215

[B26] Plancher G. Nicolas S. Piolino P. (2008). “Virtual (reality) as a tool for assessing episodic memory,” in *Proceedings of the 2008 ACM Symposium on Virtual Reality Software and Technology* (New York, NY: ACM), 179–182. 10.1145/1450579.1450617

[B27] Plancher G. Tirard A. Gyselinck V. Nicolas S. Piolino P. (2012). Using virtual reality to characterize episodic memory profiles in amnestic mild cognitive impairment and Alzheimer’s disease: influence of active and passive encoding. *Neuropsychologia* 50 592–602. 10.1016/j.neuropsychologia.2011.12.013 22261400

[B28] Raffard S. D’Argembeau A. Bayard S. Boulenger J. P. Van der Linden M. (2010). Scene construction in schizophrenia. *Neuropsychology* 24 608–615. 10.1037/a0019113 20804249

[B29] Reid L. M. Maclullich A. M. J. (2006). Subjective memory complaints and cognitive impairment in older people. *Dement. Geriatr. Cogn. Disord.* 22 471–485. 10.1159/000096295 17047326

[B30] Repetto C. Serino S. Macedonia M. Riva G. (2016). Virtual reality as an embodied tool to enhance episodic memory in elderly. *Front. Psychol.* 7:1839. 10.3389/fpsyg.2016.01839 27909424 PMC5113123

[B31] Rice H. J. Rubin D. C. (2009). I can see it both ways: first- and third-person visual perspectives at retrieval. *Conscious. Cogn.* 18 877–890. 10.1016/J.CONCOG.2009.07.004 19692271 PMC2784183

[B32] Riva G. Mantovani F. (2012). From the body to the tools and back: a general framework for presence in mediated interactions. *Interact. Comput.* 4 203–210. 10.1016/j.intcom.2012.04.007

[B33] Riva G. Waterworth J. A. Waterworth E. L. (2004). The layers of presence: a bio-cultural approach to understanding presence in natural and mediated environments. *Cyberpsychol. Behav. Soc. Netw.* 7 402–416. 10.1089/cpb.2004.7.402 15331027

[B34] Riva G. Waterworth J. A. Waterworth E. L. Mantovani F. (2011). From intention to action: the role of presence. *New Ideas Psychol.* 29 24–37. 10.1016/j.newideapsych.2009.11.002

[B35] Rizzo A. A. Schultheis M. Kerns K. A. Mateer C. (2004). Analysis of assets for virtual reality applications in neuropsychology. *Neuropsychol. Rehabil.* 14 207–239. 10.1080/09602010343000183

[B36] Robertson C. E. Hermann K. L. Mynick A. Kravitz D. J. Kanwisher N. (2016). Neural representations integrate the current field of view with the remembered 360° panorama in scene-selective cortex. *Curr. Biol.* 26 2463–2468. 10.1016/j.cub.2016.07.002 27618266

[B37] Rosci C. Sacco D. Laiacona M. Capitani E. (2005). Interpretation of a complex picture and its sensitivity to frontal damage: a reappraisal. *Neurol. Sci.* 25 322–330. 10.1007/s10072-004-0365-6 15729495

[B38] Rubin D. C. Umanath S. (2015). Event memory: a theory of memory for laboratory, autobiographical, and fictional events. *Psychol. Rev.* 122 1–23. 10.1037/a0037907 25330330 PMC4295926

[B39] Serino S. Baglio F. Rossetto F. Realdon O. Cipresso P. Parsons T. D. (2017). Picture interpretation test (PIT) 360°: an innovative measure of executive functions. *Sci. Rep.* 7 1–10. 10.1038/s41598-017-16121-x 29167494 PMC5700040

[B40] Slater M. Sanchez-Vives M. V. (2016). Enhancing our lives with immersive virtual reality. *Front. Robot. AI* 3:74 10.3389/frobt.2016.00074

[B41] Slobounov S. M. Ray W. Johnson B. Slobounov E. Newell K. M. (2015). Modulation of cortical activity in 2D versus 3D virtual reality environments: an EEG study. *Int. J. Psychophysiol.* 95 254–260. 10.1016/j.ijpsycho.2014.11.003 25448267

[B42] Spiers H. J. Burgess N. Maguire E. A. Baxendale S. A. Hartley T. Thompson P. J. (2001). Unilateral temporal lobectomy patients show lateralized topographical and episodic memory deficits in a virtual town. *Brain* 124 2476–2489. 10.1093/brain/124.12.2476 11701601

[B43] Spinnler H. Tognoni G. (1987). Italian group on the neuropsychological study of ageing: Italian standardization and classification of neuropsychological tests. *Ital. J. Neurol. Sci.* 6(Suppl. 8), 1–120. 3330072

[B44] Suddendorf T. Addis D. Corballis M. (2009). “Mental time travel and the shaping of the human mind. *Philos. Trans. R. Soc. Lond. B Biol. Sci.* 364 1317–1324. 10.1098/rstb.2008.0301 19528013 PMC2666704

[B45] Sutcliffe A. Gault B. Shin J.-E. (2005). Presence, memory and interaction in virtual environments. *Int. J. Hum. Comput. Stud.* 62 307–327. 10.1016/j.ijhcs.2004.11.010

[B46] Tulving E. (1983). *Elements of Episodic Memory.* Oxford: Clarendon Press.

[B47] Tulving E. (2002) Episodic memory: from mind to brain. *Annu. Rev. Psychol.* 53 1–25. 10.1146/annurev.psych.53.100901.135114 11752477

[B48] Tulving E. Markowitsch H. J. (1998). Episodic and declarative memory: role of the hippocampus. *Hippocampus* 8 198–204. 10.1002/(SICI)1098-1063(1998)8:3<198::AID-HIPO2>3.0.CO;2-G 9662134

[B49] Tulving E. Murray D. (1985). Elements of episodic memory. *Can. Psychol.* 26 235–238. 10.1037/h0084438

[B50] Wilson B. Cockburn J. Baddeley A. (1986). *The Rivermead Behavioural Memory Test (RBMT).* Bury St Edmunds: Thames Valley TestCompany.

[B51] Wilson B. Cockburn J. Baddeley A. Hiorns R. (1989). The development and validation of a test battery for detecting and monitoring everyday memory problems. *J. Clin. Exp. Neuropsychol.* 11 855–870. 10.1080/01688638908400940 2592527

